# A sarcopenia index based on serum creatinine and cystatin C cannot accurately detect either low muscle mass or sarcopenia in urban community-dwelling older people

**DOI:** 10.1038/s41598-018-29808-6

**Published:** 2018-08-01

**Authors:** Qian He, Jiaojiao Jiang, Lingling Xie, Luoying Zhang, Ming Yang

**Affiliations:** 1 Outpatient Department, West China Hospital, Sichuan University, No. 37 Guoxue Lane, Chengdu, Sichuan China; 2 The Center of Rehabilitation, West China Hospital, Sichuan University, No. 37 Guoxue Lane, Chengdu, Sichuan China; 3 The Center of Gerontology and Geriatrics, West China Hospital, Sichuan University, No. 37 Guoxue Lane, Chengdu, Sichuan China; 4 The Health Management Center, Shangjin Nanfu Hospital, No. 253 Shangjin Street, Chengdu, Sichuan China

## Abstract

The aim of this study was to evaluate the diagnostic value of the sarcopenia index (serum creatinine [mg/dl]/cystatin C [mg/dl] × 100) for estimating low muscle mass and sarcopenia in community-dwelling older adults. We included 371 older adults (≥60 years) with normal kidney function. Four common diagnostic criteria (the European Working Group on Sarcopenia in Older People (EWGSOP), Asia Working Group for Sarcopenia (AWGS), International Working Group on Sarcopenia (IWGS), and Foundation for the National Institutes of Health (FNIH) criteria) were separately applied as the “gold standard”. The receiver operating characteristic (ROC) curves and the area under the ROC curves (AUC) were applied to evaluate the overall diagnostic accuracy. For identifying low muscle mass, the AUC ranged from 0.505 (95% confidence interval [CI] 0.453–0.557) to 0.558 (95% CI 0.506–0.609). For identifying sarcopenia, the AUC ranged from 0.555 (95% CI 0.503–0.606) to 0.618 (95% CI 0.566–0.668). Subgroup analyses according to gender showed similar results. In conclusion, the sarcopenia index based on serum creatinine and cystatin C may not serve as biomarkers of either low muscle mass or sarcopenia in urban community-dwelling older people with normal kidney function.

## Introduction

Sarcopenia refers to an aging-related loss of muscle mass and function^1^. It is prevalent in older people, especially in the elders who live in long-term care facilities or hospitals^2^. Recently, clinicians in other disciplines, such as oncologists^3^, cardiologists^4^, and respirologists^5^, in addition to gerontologists and geriatricians, have recognized the important role of sarcopenia in the prognosis of their patients.

According to current international consensuses, the diagnosis of sarcopenia should include low muscle mass (usually measured by computed tomography [CT], magnetic resonance imaging [MRI], dual-energy X-ray analysis [DXA], or bioelectrical impedance analysis [BIA]), low muscle strength (usually measured by handgrip strength), and/or low physical performance (usually measured by gait speed)^6–9^. In other words, the diagnosis of sarcopenia requires special medical devices, which are unfeasible, expensive, or carry radiation risk. On the other hand, individuals who cannot perform a walking test are actually at high risk of sarcopenia; however, these individuals must be excluded from the sarcopenia studies according to current diagnostic algorithms^10^. Therefore, several attempts have been made to identify sarcopenia based on self-reported questionnaires (e.g., SARC-F (sluggishness, assistance in walking, rise from a chair, climb stairs, falls))^11^ or serum biomarkers^11,12^.

Recently, a novel sarcopenia index was developed by Kashani *et al*.^12^. This index is calculated using the following equation: serum creatinine (mg/dl)/serum cystatin C (mg/dl) × 100. Serum creatinine and cystatin C are generated by skeletal muscle mass and all nucleated cells, respectively and both biomarkers are eliminated via the kidneys. Therefore, in individuals with normal kidney function, this index was supposed to be useful to estimate skeletal muscle mass^12^. In their study, Kashani *et al*. reported that the sarcopenia index could estimate muscle mass and predict in-hospital mortality and 90-day mortality in ICU patients^12^. This tool must be internally and externally validated in different study populations. Most recently, the same research group further reported that this sarcopenia index was significantly correlated with CT scan-measured muscle surface area at the L2 and L3 vertebral levels in a small group of lung transplant candidates^13^.

Serum creatinine and cystatin C are regular tests in older adults. Therefore, if the sarcopenia index can estimate sarcopenia in older adults, the screening of sarcopenia in the elderly would be significantly simplified. Thus, we hypothesized that the sarcopenia index may be a potentially useful tool for estimating low muscle mass or sarcopenia in older people with normal kidney function. The aim of this study was to evaluate the diagnostic accuracy of the sarcopenia index for detecting low muscle mass and sarcopenia in community-dwelling older people.

## Methods

### Study population

We conducted a diagnostic accuracy study. From October to November 2017, we consecutively recruited older people aged ≥60 years who were living in an urban community in Chengdu, China, and agreed to sign a written informed consent form. We recruited these participants using a convenient sampling method through posters and WeChat (the most popular social media-app in China). Individuals with the following conditions were excluded: (1) severe mental diseases; (2) a pacemaker; (3) unable to walk or communicate with study staff; (4) clinically visible edema; (5) severe heart failure; (6) estimated glomerular filtration rate (eGFR) <90 mL/min/1.73 m^2^. The participant information, anthropometric measurements, and the measurements of skeletal muscle mass, gait speed test, and handgrip strength test were performed on the same day by trained nurses. The Research Ethics Committee of Sichuan University approved the study protocol. All methods in this study were in accordance with relevant regulations and guidelines.

### Sarcopenia index

A fasting blood sample was obtained from each participant by experienced nurses in the morning after at least an 8-h fast. There was no food intake restriction on the day before the blood sampling. Serum creatinine and cystatin C were measured using the Roche enzymatic method (Creatinine Jaffe Gen.2 assay: Roche Diagnostics GmbH, Mannheim, Germany) and an immunoturbidimetric assay (Sichuan Mike Biotechnology Co., Ltd., Chengdu, China), respectively. Sarcopenia index = serum creatinine (mg/dl)/serum cystatin C (mg/dl) × 100.

### Measurement of skeletal muscle mass

A trained nurse applied a bioelectrical impedance analysis (BIA) device (InBody 230, Biospace Co., Ltd., Korea) to measure the appendicular skeletal muscle mass (ASM) and body fat mass of each participant. The skeletal muscle mass (SMI) was then calculated based on the equation: SMI (kg/m^2^) = ASM/height^2^.

### Measurement of gait speed

A trained nurse asked each participant to walk a 4-m course at their usual walking speed and recorded the time of completing the course. Gait speed (m/s) was then calculated by 4 m/time consumed in (s).

### Measurement of handgrip strength

A trained nurse applied a handheld dynamometer based on strain gauge sensors (EH101, Xiangshan Inc., Guangdong, China) to measure the handgrip strength of each participant. Each hand was tested three times and the highest value was recorded.

### Assessment of low muscle mass and sarcopenia using different criteria

Due to the lack of a unique “gold standard” criteria for either low muscle mass or sarcopenia, we separately applied the four common international criteria as the reference standard: (1) the European Working Group on Sarcopenia in Older People (EWGSOP)^6^; (2) the International Working Group on Sarcopenia (IWGS)^9^; (3) the Asia Working Group for Sarcopenia (AWGS)^7^; and (4) the Foundation for the National Institutes of Health (FNIH) Sarcopenia Project^8^. Detailed information on cut-off points and criteria are provided in Table 1.

**Table 1:** Diagnostic criteria for sarcopenia in this study.

	① Low muscle mass	② Low handgrip strength (kg)	③ Low gait speed (m/s)	Diagnostic criteria
EWGSOP	SMI < 6.28 kg/m^2^ for men; SMI < 5.08 kg/m^2^ for women^†^;	<30 for men; <20 for women;	< 0.8 for both genders	① + ② or ① + ③
IWGS	SMI ≤ 7.23 kg/m^2^ for men; SMI ≤ 5.67 kg/m^2^ for women;	—	<1.0 for both genders	① + ③
FNIH	ASM/BMI < 0.789 for men; ASM/BMI < 0.512 for women;	<26 for men; <16 for women;	<0.8 for both genders	① + ② + ③
AWGS	<7.0 kg/m^2^ for men; <5.7 kg/m^2^ for women;	<26 for men; <18 for women;	<0.8 for both genders	① + ② or ① + ③

ASM, appendicular skeletal muscle mass; AWGS, Asia Working Group for Sarcopenia; BMI, body mass index; EWGSOP, European Working Group on Sarcopenia in Older People; FNIH, Foundation for the National Institutes of Health; IWGS, International Working Group on Sarcopenia; SMI, skeletal muscle index.

^†^The cut-off points were based on the lowest quintile values of the distribution of our study population.

### Covariates

A trained nurse measured the body weight and height of each participant with a digital floor scale and a stadiometer, respectively. Next, a body mass index (BMI) was calculated using the equation: BMI (kg/m^2^) = body weight/height^2^. In addition, trained nurses collected the following information through face-to-face interviews: age, gender, and the medical history of the following chronic diseases: hypertension, coronary heart disease, diabetes, chronic obstructive pulmonary disease, and stroke.

### Statistical analyses

All statistical analyses in this study were performed in MedCalc Statistical Software 15.2 (MedCalc Software bvba, Ostend, Belgium). All statistical tests were two-sided, and a p-value of <0.05 indicated statistical significance.

Categorical data were presented as numbers (percentage). Continuous data with a normal distribution or a skewed distribution were presented as the mean (standard deviation [SD]) or median (minimum-maximum), respectively. To compare the differences between groups, the chi-squared test, one-way analysis of variance (ANOVA), and Mann-Whitney test were applied for categorical data, continuous data with a normal distribution, and continuous data with a skewed distribution, respectively.

We applied a receiver operating characteristics (ROC) curve to evaluate the overall accuracy of the sarcopenia index. The area under the ROC curve (AUC) and 95% confidence interval (CI) were calculated^14^. Because men generally have a larger muscle mass than women and the prevalence of sarcopenia was not equivalent between men and women^15^, we also performed subgroup analyses according to gender.

## Results

### Characteristics of the study population

A total of 152 men and 219 women were included (mean age: 72.9 ± 6.0 versus 70.8 ± 5.5, respectively, p = 0.039). Table 2 shows the characteristics of our study population. The sarcopenia index was significantly higher in men than in women (p < 0.001).

**Table 2:** Characteristics of the study population.

Characteristics	Total (n = 371)	Men (n = 152)	Women (n = 219)	P
Age (years)^‡^	71.4 (5.8)	72.9 (6.0)	70.8 (5.5)	0.039
**Chronic diseases** ^†^				
Hypertension	110 (29.6)	46 (30.3)	64 (29.2)	0.829
Coronary heart disease	32 (8.6)	10 (6.6)	22 (10.0)	0.242
Diabetes	33 (8.9)	15 (9.9)	18 (8.2)	0.583
Stroke	46 (12.4)	13 (8.6)	33 (15.1)	0.061
COPD	30 (8.1)	13 (8.6)	17 (7.8)	0.784
BMI (kg/m^2^)^‡^	24.2 (3.3)	24.1 (3.3)	24.3 (3.3)	0.602
Gait speed (m/s)^‡^	0.88 (0.23)	0.92 (0.26)	0.86 (0.21)	0.013
Handgrip strength (kg)^‡^	22.9 (8.9)	29.6 (8.9)	18.3 (5.4)	<0.001
ASM (kg)^‡^	14.9 (3.7)	18.2 (3.0)	12.6 (2.2)	<0.001
Body fat mass^‡^	19.0 (5.6)	18.1 (6.0)	19.6 (5.3)	0.010
Sarcopenia index^∗^	81 (41–193)	88.1 (53–193)	74.8 (41–124)	<0.001
Low muscle mass based on EWGSOP criteria	72 (19.4)	31 (20.4)	41 (18.7)	0.689
Low muscle mass based on AWGS criteria	196 (52.8)	82 (53.9)	114 (52.1)	0.720
Low muscle mass based on IWGS criteria	210 (56.6)	100 (65.8)	110 (50.2)	0.003
Low muscle mass based on FNIH criteria	205 (55.3)	97 (63.8)	108 (49.3)	0.006
Sarcopenia based on EWGSOP criteria	42 (11.3)	16 (10.5)	26 (11.9)	0.687
Sarcopenia based on AWGS criteria	57 (15.4)	18 (11.8)	39 (17.8)	0.117
Sarcopenia based on IWGS criteria	91 (24.5)	37 (24.3)	54 (24.7)	0.945
Sarcopenia based on FNIH criteria	55 (14.8)	22 (14.5)	33 (15.1)	0.874

^†^Data are presented as n (%). ^‡^Data are presented as mean (standard deviation). ^∗^Data are presented as median (interquartile range).

ASM, appendicular skeletal muscle mass; AWGS, the Asian Working Group for Sarcopenia; BMI, body mass index; CC, calf circumference; COPD, chronic obstructive pulmonary disease; EWGSOP, the European Working Group on Sarcopenia in Older People; FNIH, the Foundation for the National Institutes of Health; IWGS, the International Working Group on Sarcopenia.

### Prevalence of low muscle mass and sarcopenia

According to the EWGSOP, AWGS, IWGS and FNIH criteria, the prevalence of low muscle mass in the entire study population ranged from 19.4% to 56.6%, whereas the prevalence of sarcopenia ranged from 11.3% to 24.5% (Table 2). Despite the diagnostic criteria, the prevalence of sarcopenia was not significantly different between men and women (Table 2). However, when using the IWGS or FNIH criteria, the prevalence of low muscle mass was significantly higher in men than in women (Table 2).

### The diagnostic accuracy of the sarcopenia index for identifying low muscle mass

**Figure 1:**
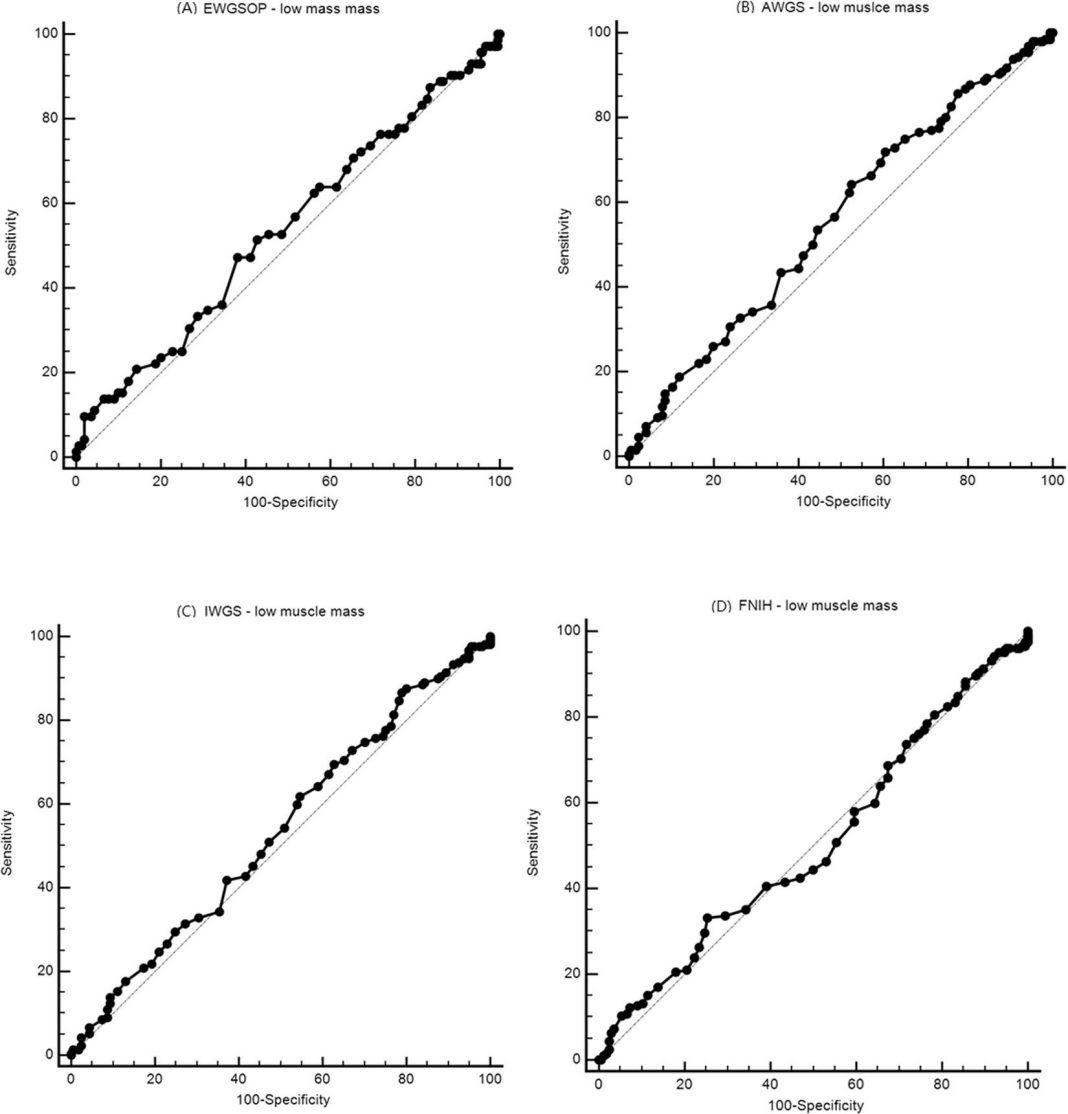
The ROC curves of the sarcopenia index for estimating low muscle mass against different “gold standards” in the entire study population: (**A**) EWGSOP criteria; (**B**) AWGS criteria; (**C**) IWGS criteria; and (**D**) FNIH criteria.

Table 3 shows the results of sensitivity/specificity analysis and the AUC of the sarcopenia index for identifying low muscle mass using different diagnostic criteria as the “gold standard”. Figure 1 shows the ROC curves of the sarcopenia index for identifying low muscle mass against different “gold standards” in the entire study population. The AUC of the sarcopenia index ranged from 0.505 (95% CI 0.453–0.557) to 0.558 (95% CI 0.506–0.609) (Table 3). The subgroup analyses showed similar results in men (Table 3 and Supplementary Fig. 1) and women (Table 3 and Supplementary Fig. 2).

**Table 3:** Sensitivity/specificity analyses and ROC models for the sarcopenia index validation against different criteria of low muscle mass or sarcopenia.

	Low muscle mass	Sarcopenia
	AUC	AUC
Total		
EWGSOP	0.538 (0.486–0.589)	0.618 (0.566–0.668)
AWGS	0.558 (0.506–0.609)	0.595 (0.543–0.645)
IWGS	0.534 (0.481–0.585)	0.555 (0.503–0.606)
FNIH	0.505 (0.453–0.557)	0.555 (0.503–0.606)
Men		
EWGSOP	0.569 (0.486–0.649)	0.689 (0.609–0.762)
AWGS	0.609 (0.527–0.687)	0.620 (0.538–0.698)
IWGS	0.657 (0.575–0.732)	0.581 (0.498–0.660)
FNIH	0.529 (0.446–0.610)	0.591 (0.509–0.670)
Women		
EWGSOP	0.531 (0.463–0.599)	0.582 (0.514–0.648)
AWGS	0.547 (0.478–0.614)	0.557 (0.488–0.624)
IWGS	0.551 (0.483–0.619)	0.566 (0.498–0.633)
FNIH	0.563 (0.494–0.630)	0.554 (0.486–0.621)

Data are presented as 95% confidence interval in parentheses.

AWGS, the Asian Working Group for Sarcopenia; AUC, area under the curve; EWGSOP, the European Working Group on Sarcopenia in Older People; FNIH, the Foundation for the National Institutes of Health; IWGS, the International Working Group on Sarcopenia.

### The diagnostic accuracy of the sarcopenia index for identifying sarcopenia

Similarly, the AUC of the sarcopenia index for identifying sarcopenia against different “gold standards” ranged from 0.555 (95% CI 0.503–0.606) to 0.618 (95% CI 0.566–0.668) (Table 3 and Supplementary Fig. 3). The subgroup analyses showed similar results in men (Supplementary Fig. 4) and women (Supplementary Fig. 5).

## Discussion

Generally, an AUC of >0.9 indicates high accuracy, 0.7 to 0.9 indicates moderate accuracy, 0.5 to 0.7 indicates low accuracy, and 0.5 indicates chance results^14^. In this study, the AUC of the sarcopenia index ranged from 0.505 to 0.558 for detecting low muscle mass and from 0.555 to 0.618 for detecting sarcopenia when using different “gold standards”. These results indicated that the sarcopenia index could not accurately detect either low muscle mass or sarcopenia in our study population.

Although sarcopenia was originally defined as aging-related loss of muscle mass, there is currently an agreement that the definition of sarcopenia should include not only low muscle mass but also low muscle strength and/or physical performance^16,17^. Both previous studies, which validated the sarcopenia index in ICU patients^12^ and lung transplant candidates^13^, defined sarcopenia only according to muscle mass. Therefore, we evaluated the diagnostic value of the sarcopenia index for identifying both low muscle mass and sarcopenia in this study.

The sarcopenia index was originally designed to estimate muscle mass instead of muscle function^13^. Previous studies have reported that the sarcopenia index was significantly related to CT scan-measured muscle mass at the L4 vertebral level in ICU patients and at the L2 and L3 vertebral levels in lung transplant candidates^12,13^. However, our study demonstrated that the diagnostic value of the sarcopenia index was limited to only focusing on low muscle mass. The methods for measuring muscle mass may partly contribute to the difference between the results of our study and those of the two previous studies. CT is a gold standard for estimating muscle mass^6^, but the cost, availability, and X-ray exposure make this technique unsuitable for community-dwelling older people. Our study applied a BIA device to estimate muscle mass. BIA has been suggested to be a good portable alternative method by EWGSOP and AWGS^6,7^. Further, a previous study demonstrated that CT-measured muscle mass at the level of L3 is closely correlated with whole-body BIA (r = 0.83)^18^.

It is noteworthy that the sarcopenia index was developed based on young critically ill patients^12^. The correlation coefficient of the sarcopenia index with the muscle mass measured by CT was not very strong^12^. Although the sarcopenia index was validated in a study population with lung transplantation^13^. The sample size was only 28, and the Pearson correlation coefficient was weak^13^. Our study also indicated that the sarcopenia index was not suitable for predicting muscle mass or sarcopenia. Therefore, the sarcopenia index may be a questionable biomarker of muscle mass. The ideal method would be to calculate the sarcopenia index in young healthy adults and compare it with elderly adults to determine the cut-offs and quantify the muscle mass.

One of the major limitations of our study is that we did not assess the value of the sarcopenia index for predicting clinically important health outcomes. Kashani *et al*. reported that the sarcopenia index could predict in-hospital mortality and 90-day mortality in ICU patients^12^. Another recent study found that the creatinine/cystatin C ratio could predict the toxicity of chemotherapy in patients with non-small cell lung cancer^19^. Moreover, Tsuneda *et al*. reported that the creatinine/cystatin C ratio was associated with unfavorable prognosis due to all causes in older patients^20^. Second, we only included older people living in an urban community; therefore, our results may not represent those living in rural or semirural areas. Third, the relatively small sample size might contribute to the negative results.

## Conclusion

The sarcopenia index based on serum creatinine and cystatin C cannot accurately detect either low muscle mass or sarcopenia in urban community-dwelling older people with normal kidney function when using four common criteria as the “gold standards”. Further studies are needed to evaluate the diagnostic value of this sarcopenia index for estimating sarcopenia in older people in different settings. The prognostic value of the sarcopenia index for predicting clinical outcomes in older people also warrants further study.

## Electronic supplementary material

Supplementary Figure 1

Supplementary Figure 2

Supplementary Figure 3

Supplementary Figure 4

Supplementary Figure 5
