# Supplementary material for: A sarcopenia index based on serum creatinine and cystatin C cannot accurately detect either low muscle mass or sarcopenia in urban community-dwelling older people
Source: Sci Rep. 2018 Aug 1;8:11534. doi: 10.1038/s41598-018-29808-6 (PMC6070576; doi:10.1038/s41598-018-29808-6)

Title page

Title:

A sarcopenia index based on serum creatinine and cystatin C cannot accurately detect either low muscle mass or sarcopenia in urban community-dwelling older people

Authors:

Qian He, MSN [1]; Jiaojiao Jiang, MD [2]; Lingling Xie, MSN [3]; Luoying Zhang, MSN [4]; Ming Yang, MD [3]

Running title

Sarcopenia index failed to detect sarcopenia

Affiliations and addresses of all authors:

1. Outpatient Department, West China Hospital, Sichuan University, No. 37 Guoxue Lane, Chengdu, Sichuan, China.
2. The Center of Rehabilitation, West China Hospital, Sichuan University, No. 37 Guoxue Lane, Chengdu, Sichuan, China.
3. The Center of Gerontology and Geriatrics, West China Hospital, Sichuan University, No. 37 Guoxue Lane, Chengdu, Sichuan, China.
4. The Health Management Center, Shangjin Nanfu Hospital, No. 253 Shangjin

Street, Chengdu, Sichuan, China.

Information of the corresponding author:

Ming Yang, The Center of Gerontology and Geriatrics, West China Hospital, Sichuan University, No. 37 Guoxue Lane, Chengdu, China. Phone: +86 28 8542 2326. Fax: +86 28 8542 2321. Email: yangmier@gmail.com

Supplementary Figure 3. The ROC curves of the sarcopenia index for estimating sarcopenia against different "gold standards" in the whole study population: (A) EWGSOP criteria; (B) AWGS criteria; (C) IWGS criteria; and (D) FNIH criteria.

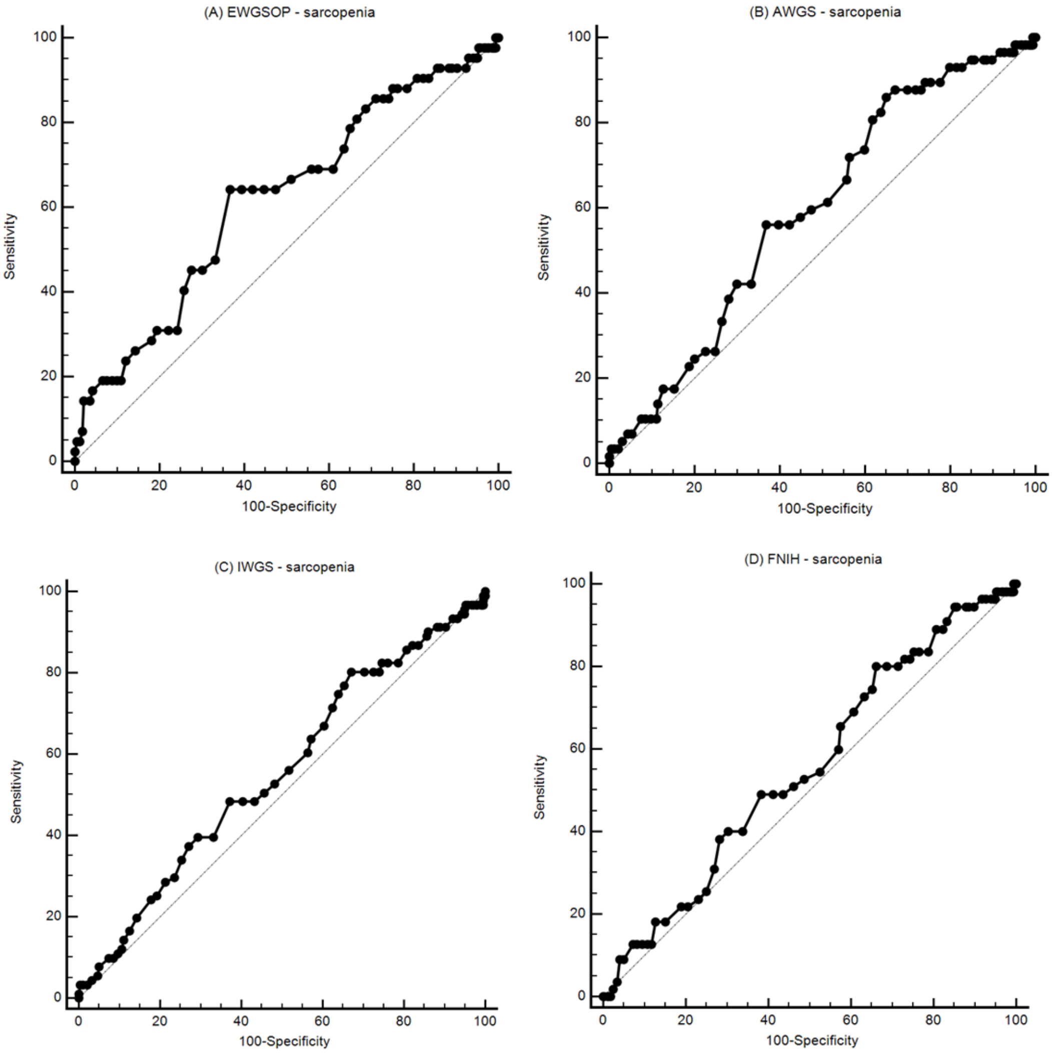

Supplement: Supplementary file 3 — Supplementary Figure 3 [file 41598_2018_29808_MOESM3_ESM.pdf]
